# Complication Differences Between the Tumescent and Non-Tumescent Dissection Techniques for Mastectomy: A Meta-Analysis

**DOI:** 10.3389/fonc.2021.648955

**Published:** 2022-01-10

**Authors:** Yi Yang, Juanying Zhu, Xinghua Qian, Jingying Feng, Fukun Sun

**Affiliations:** ^1^ Department of Breast Surgery, Jiaxing Maternity and Child Health Care Hospital, Affiliated Women and Children’s Hospital of Jiaxing University, Jiaxing, China; ^2^ Department of Anesthesia, Jiaxing Maternity and Child Health Care Hospital, Affiliated Women and Children’s Hospital of Jiaxing University, Jiaxing, China; ^3^ Department of Nursing, Jiaxing Maternity and Child Health Care Hospital, Affiliated Women and Children’s Hospital of Jiaxing University, Jiaxing, China

**Keywords:** breast cancer, mastectomy, epinephrine, lignocaine, complications, skin necrosis

## Abstract

**Purpose:**

We conducted a systematic literature search and pooled data from studies to compare the incidence of complications between the tumescent and non-tumescent techniques for mastectomy.

**Methods:**

We searched PubMed, Embase, BioMed Central, Ovid, and CENTRAL databases for studies comparing the two mastectomy techniques up to November 1^st^, 2020. We used a random-effects model to calculate odds ratios (OR) with 95% confidence intervals (CI).

**Results:**

Nine studies were included with one randomized controlled trial (RCT). Meta-analysis indicated no statistically significant difference in the incidence of total skin necrosis (OR 1.18 95% CI 0.71, 1.98 I^2^ = 82% p=0.52), major skin necrosis (OR 1.58 95% CI 0.69, 3.62 I^2^ = 71% p=0.28), minor skin necrosis (OR 1.11 95% CI 0.43, 2.85 I^2^ = 72% p=0.83), hematoma (OR 1.19 95% CI 0.80, 1.79 I^2^ = 4% p=0.39), and infections (OR 0.87 95% CI 0.54, 1.40 I^2^ = 54% p=0.56) between tumescent and non-tumescent groups. Analysis of studies using immediate alloplastic reconstruction revealed no statistically significant difference in the incidence of explantation between the two groups (OR 0.78 95% CI 0.46, 1.34 I^2^ = 62% p=0.37). Multivariable-adjusted ORs on total skin necrosis were available from three studies. Pooled analysis indicated no statistically significant difference between tumescent and non-tumescent groups (OR 1.72 95% CI 0.72, 4.13 I^2^ = 87% p=0.23).

**Conclusion:**

Low-quality evidence derived mostly from non-randomized studies is indicative of no difference in the incidence of skin necrosis, hematoma, seroma, infection, and explantation between the tumescent and non-tumescent techniques of mastectomy. There is a need for high-quality RCTs to further strengthen the evidence.

## Introduction

Since the first description of the tumescent dissection mastectomy method by Worland (1) in 1996, the technique has gained popularity for both breast cancer and esthetic surgical procedures (2, 3). Tumescent dissection involves an injection of a very dilute solution of local anesthetic with epinephrine and a crystalloid into the subcutaneous tissues of the breast (4) using multiple small stab punctures. The solution is injected just before the initial incision thereby creating tension between the anatomical planes and hydro-dissecting the tissues. The space created by the solution enhances visibility and ease of dissection, and allows the surgeon to distinguish between the subcutaneous and glandular tissues (5). Dissection can be easily carried out using sharp scissors obviating the need for electrocautery near the skin flaps which might lead to soft tissue damage by the dissipating thermal energy (6). The epinephrine in the mixture causes vasoconstriction, which is further enhanced by the tamponading effect of the high volume infiltration on the sub-dermal vessels (7). Another potential advantage is the analgesic effect offered by the local anesthetic which has been confirmed by researchers (8, 9).

However, despite the technique’s benefits, the risk of skin flap necrosis with the use of the tumescent solution is disconcerting to many surgeons. Skin flap necroses after mastectomy are a serious complication leading to patient dissatisfaction and increased healthcare costs (10). More importantly, they can cause a delay in the initiation of adjuvant therapies after surgery thereby affecting patient outcomes (11). In this context, clarifying the impact of the tumescent dissection technique vis-a-vis the standard surgical technique on the incidence of postsurgical complications in patients undergoing mastectomies is important. In a systematic review and meta-analysis by Siotos et al (3), authors found that the use of the tumescent technique in mastectomies is associated with a significantly increased risk of skin necrosis. However, as pointed out in the study itself, they were only able to pool data from five studies. Thus, we conducted an updated systematic literature search and pooled data from studies to strengthen the published evidence by comparing the incidence of complications between the tumescent and non-tumescent surgical techniques for mastectomies.

## Materials and Methods

### Search Strategy

Two independent reviewers carried out a comprehensive electronic search of PubMed, Embase, BioMed Central, Ovid, and CENTRAL databases without language restrictions. The search was conducted from the inception of these databases to the 1^st^ of November, 2020. Various combinations of the following search terms were included in the database search: “breast surgery”, “mastectomy”, “hydrodissection”, “tumescent”, “lignocaine”, “epinephrine” and “local anaesthetic”. The two researchers reviewed the titles and abstracts of the articles after the database search to identify the relevant articles. They evaluated the full-text of these articles for final inclusion in the study; selection process disagreements were resolved by discussion. Finally, we also performed a manual search of the bibliography of studies meeting the inclusion criteria and of previous reviews on the topic for any missed references. We followed the PRISMA (Preferred Reporting Items for Systematic Reviews and Meta-analyses) statement guidelines during the conduct of this review (12), and we present the search strategy and results in **Supplementary Table S1** accordingly.

### Inclusion and Exclusion Criteria

We defined the inclusion and exclusion criteria of the review *a priori* based on the PICOS (Population, Intervention, Comparison, Outcome, Study type) framework as follows:

*Population*: Studies conducted on adult women undergoing mastectomy with or without immediate reconstruction.

*Intervention*: Use of tumescent dissection technique.

*Comparison*: Use of non-tumescent technique (defined as the standard surgical technique with electrocautery and/or harmonic scalpel).

*Outcomes:* Studies reporting data on complications (including skin necrosis, hematoma, infections, seroma, *etc*) after the relevant surgical procedures.

*Study type*: Randomized controlled trials (RCTs), prospective or retrospective cohort studies.

Our exclusion criteria were the following: 1) Studies on other patients (not undergoing mastectomy). 2) Non-comparative studies. 3) Studies lacking relevant outcomes. 4) Case reports and review articles.

### Data Extraction and Quality Assessment

We prepared a data extraction form beforehand to process relevant data. Information Two authors independently sourced the information, and they extracted the name of the first author, publication year, study type, study location, surgery type, non-tumescent technique used, sample size, number of breasts in the population, age of patients, proportion of smokers, proportion of diabetics, use of radiation therapy, mastectomy weight, follow-up length, and study outcomes. The outcomes of interest were the incidences of total skin necrosis, major skin necrosis, minor skin necrosis, hematoma, seroma, infections, and explantation or conversion to autologous reconstruction in cases of alloplastic reconstruction. We defined major skin necrosis as full-thickness necrosis requiring intervention in the operating room and minor skin necrosis as partial necrosis needing only local wound care. Our definition of hematoma included only those requiring surgical evacuation, and that of infections included only those requiring intravenous antibiotics with or without hospital readmission.

We assessed the quality of the studies included using the Cochrane Collaboration risk assessment tool for RCTs (13) and the risk of a bias assessment tool for non-randomized studies (RoBANS) (14). We evaluated selection of participants, confounding variables, intervention measurements, blinding of outcome assessment, incomplete outcome data, and selective outcome reporting for each study.

### Statistical Analysis

We carried out our pooled analysis using “Review Manager” (RevMan, version 5.3; Nordic Cochrane Centre [Cochrane Collaboration], Copenhagen, Denmark; 2014). On account of the inherent heterogeneity of the included studies, we chose a random-effects model for the meta-analysis of all outcomes. We calculated odds ratios (ORs) with 95% confidence intervals (CIs) to compare complications between the tumescent and non-tumescent surgical techniques. We pooled the incidence of complications per breast rather than per patient. We carried out a sub-group analysis based on the use of immediate reconstruction after the mastectomies. We also extracted the multivariable-adjusted ORs of the outcomes, if available, from the included studies. We pooled variable data if reported by at least three of the studies using the generic inverse variance model. We used the *I^2^* statistic to assess heterogeneity and classified it as low (*I^2^* values between 25% and 50%), medium (values between 50% and 75%) or high (values higher than 75%). We avoided using funnel plots to assess publication bias because each meta-analysis was based on data from more than 10 studies.

## Results

**Figure 1:**
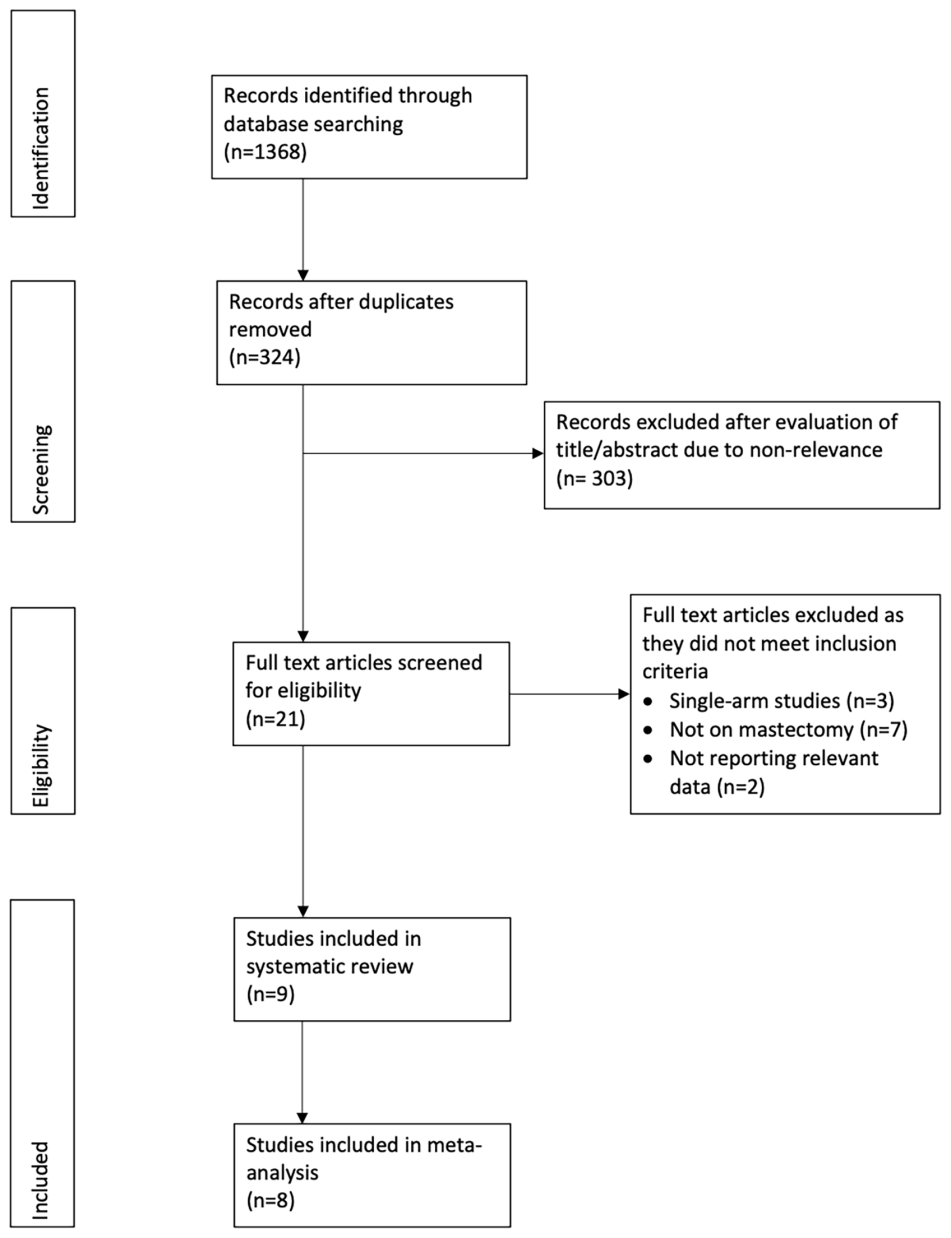
Study flowchart.

**Figure 1** shows the PRISMA flow-chart. We included nine studies in the review (15–23). **Table 1** presents their characteristics. Seven studies were retrospective, one prospective, and one an RCT. Most studies were carried out in the USA. All the patients underwent immediate reconstruction in all but in two studies. In the study of Abbott et al (21), 65.7% of patients in the tumescent group and 57.8% of patients in the non-tumescent group underwent immediate reconstruction. In the trial of Lautrup et al (15), none of the patients underwent immediate reconstruction. Two studies reported the use of a harmonic scalpel in the non-tumescent group. In the study of Khavani et al (20), the use of pre-and post-surgery radiation therapy was significantly higher in the non-tumescent group than in the tumescent group. Complication data per breast were available for all studies except for that by Gipponi et al (17), which reported data per patient. Therefore, we excluded this study from the meta-analysis. In that study, the authors reported a significantly higher incidence of minor skin necrosis in the tumescent group (2/15 patients) than in the non-tumescent group (7/15 patients) (*p*=0.45) without major skin necroses. In addition, they found no significant differences in the incidences of hematoma or wound infection between the two groups (17).

**Table 1:** Details of the studies included.

Author	Year	Study Type	Study location	Surgery type	nTT technique	Number of patients		Number of breasts		Mean age (years)		Smokers (%)		Diabetes mellitus (%)		Pre-mastectomy radiation (%)		Pre-mastectomy radiation (%)		Mean mastectomy weight (g)		Mean follow-up
						TT	nTT	TT	nTT	TT	nTT	TT	nTT	TT	nTT	TT	nTT	TT	nTT	TT	nTT	
Chun et al (23)	2011	RT	USA	Mastectomy with immediate autologous or alloplastic breast reconstruction	NR	275		100	280	46.4± 9.5	48± 9.9	30	27.1	1	2.1	7	9.3	7	5.7	625.1± 411.7	531.5± 292.1	NR
Seth et al (22)	2011	RT	USA	Mastectomy with immediate tissue expander or implant reconstruction	Electrocautery and harmonic scalpel	332	565	457	760	47.4± 9.8	48.5± 11.4	13.1	11.4	NR	NR	NR	NR	19.3	23.7	NR	NR	36.5 months
Abbott et al (21)	2012	RT	USA	Mastectomy with or without reconstruction	Electrocautery	70	64	113	88	52.5± NR	51.3± NR	5.7	9.4	NR	NR	NR	NR	NR	NR	712.4± NR	675.6± NR	NR
Khavanin et al (20)	2013	RT	USA	Mastectomy with immediate tissue expander–implant-based reconstructions	Electrocautery and harmonic scalpel	1030		890	601	47.7± 10.5	49.3± 11.1	8.88	9.15	NR	NR	2.92*	8.15*	20*	25.29*	NR	NR	21.2 months
Vargas et al (18)	2015	RT	USA	Skin- sparing mastectomy followed by immediate autologous microsurgical breast reconstruction	Electrocautery	504		336	394	49.7± 8.5	49.2± 8.1	14.6	8.4	2.4	7	21.7	18.2	NR	NR	779.4± 419.1	760.3± 409.4	NR
Gipponi et al (17)	2017	PT	Italy	Skin- sparing mastectomy or nipple-sparing mastectomy with immediate alloplastic reconstruction	NR	15	15	NR	NR	53.37	48.26	40	33	NR	NR	NR	NR	NR	NR	NR	NR	NR
Ng et al (16)	2019	RT	Canada	Nipple-sparing mastectomy with immediate alloplastic reconstruction	Electrocautery	40	22	77	39	43± 10.7	43.2± 8.7	10	9.1	NR	NR	3.9	5.1	NR	NR	326.6± 131.4	286.3± 103.4	NR
Tasoulis et al (19)	2019	RT	USA	Nipple-sparing mastectomy with immediate alloplastic reconstruction	Electrocautery	23	18	46	36	38 (25-63)^	36.5 (19-52^	0	5.6	0	0	NR	NR	NR	NR	NR	NR	6 months
Lautrup et al (15)	2020	RCT	Denmark	Mastectomy without immediate reconstruction	Electrocautery	105	98	107	102	65.6± NR	60.3± NR	16	14	8	5	0	0	NR	NR	NR	NR	NR

RT, retrospective; PT, prospective; NR, not reported; RCT, randomised controlled trial; TT, tumescent technique; nTT, non-tumescent technique; RL, Lactated Ringer’s solution.

*Statistically significant difference between TT and nTT groups as reported by the study.

^Median (Range).

### Meta-Analysis

**Figure 2:**
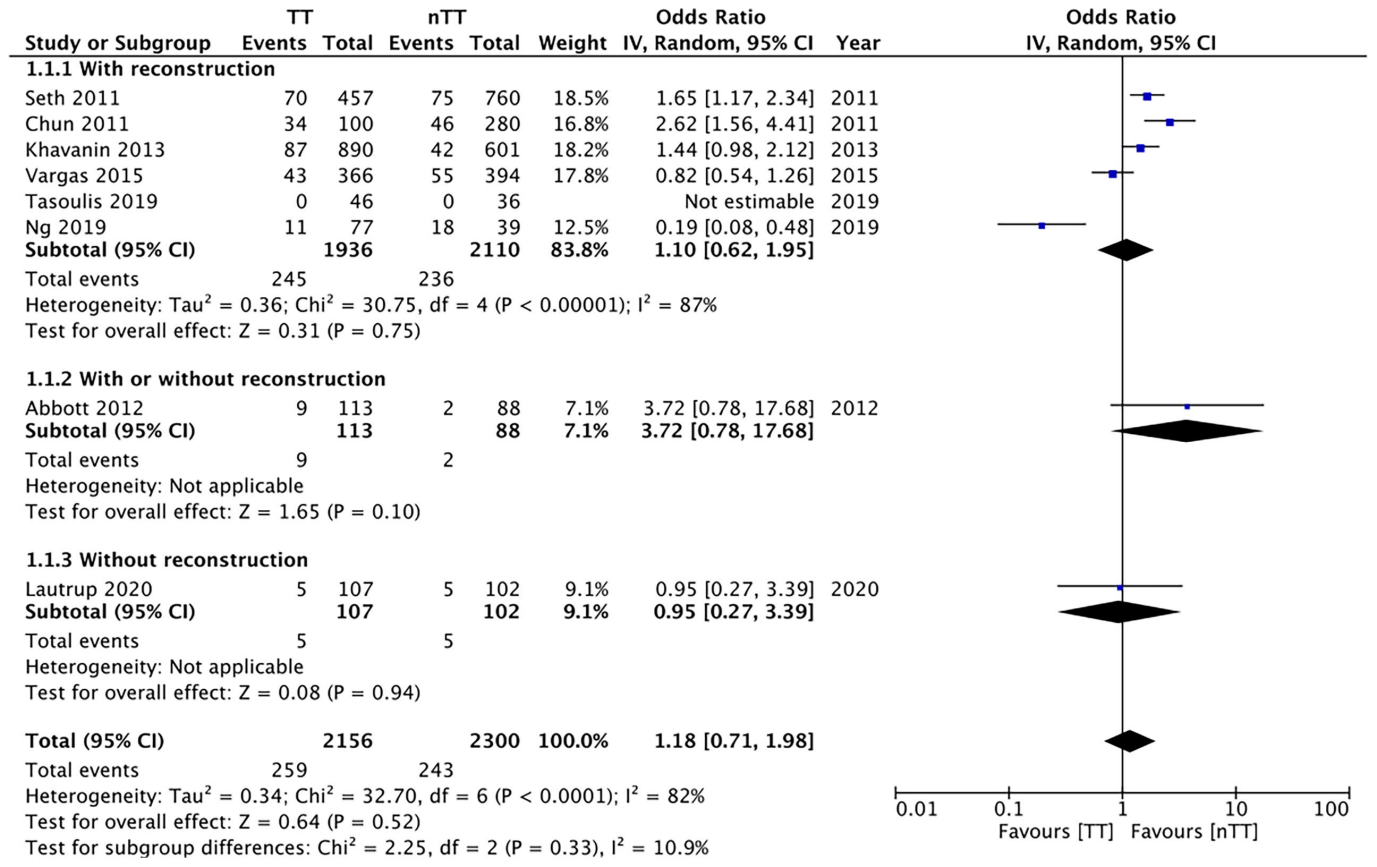
Forest plot for total skin necrosis in sub-group analysis based on immediate reconstruction. TT, tumescent group; nTT, non-tumescent group; IV, inverse variance; OR, odds ratio.

**Figure 3:**
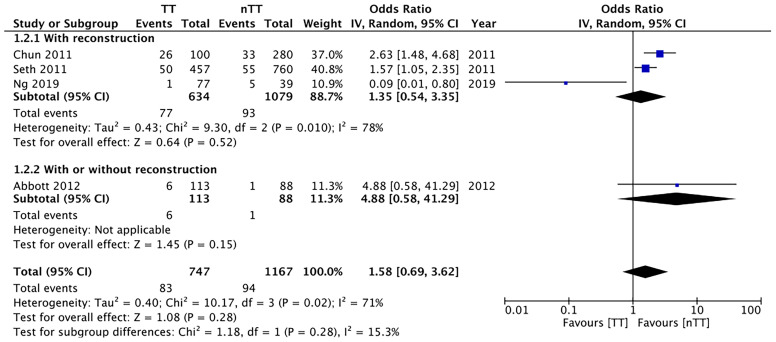
Forest plot for major skin necrosis in sub-group analysis based on immediate reconstruction. TT, tumescent group; nTT, non-tumescent group; IV, inverse variance; OR, odds ratio.

**Figure 4:**
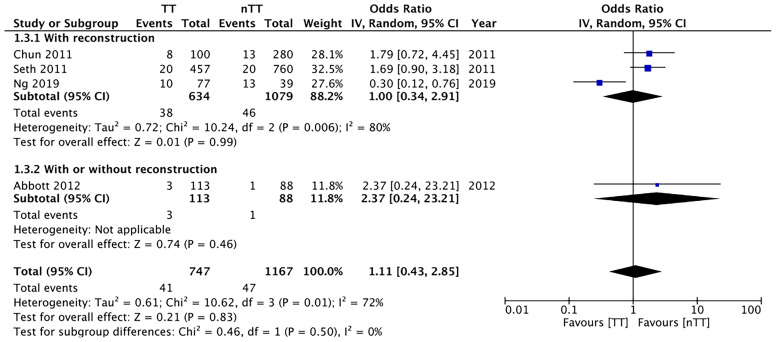
Forest plot for minor skin necrosis in sub-group analysis based on immediate reconstruction. TT, tumescent group; nTT, non-tumescent group; IV, inverse variance; OR, odds ratio.

We pooled the data on total skin necrosis from eight studies. Our meta-analysis results indicated no significant differences in the incidences of total skin necrosis between tumescent and non-tumescent groups (OR, 1.18; 95% CI, 0.71 to 1.98; *I^2^ = *82%; *p*=0.52). Our subgroup analysis based on the use of immediate reconstruction, showed similar results for all sub-groups (**Figure 2**). The incidences of major skin necrosis (OR, 1.58; 95% CI, 0.69 to 3.62; *I^2^ = *71%; *p*=0.28) and of minor skin necrosis (OR, 1.11; 95% CI, 0.43 to 2.85; *I^2^ = *72%; *p*=0.83) were also similar amongst the two study groups. The results were similar for studies using immediate reconstruction and for the study by Abott et al (21) reporting on a mixed population of patients with or without immediate reconstruction (**Figures 3**, **4**).

**Figure 5:**
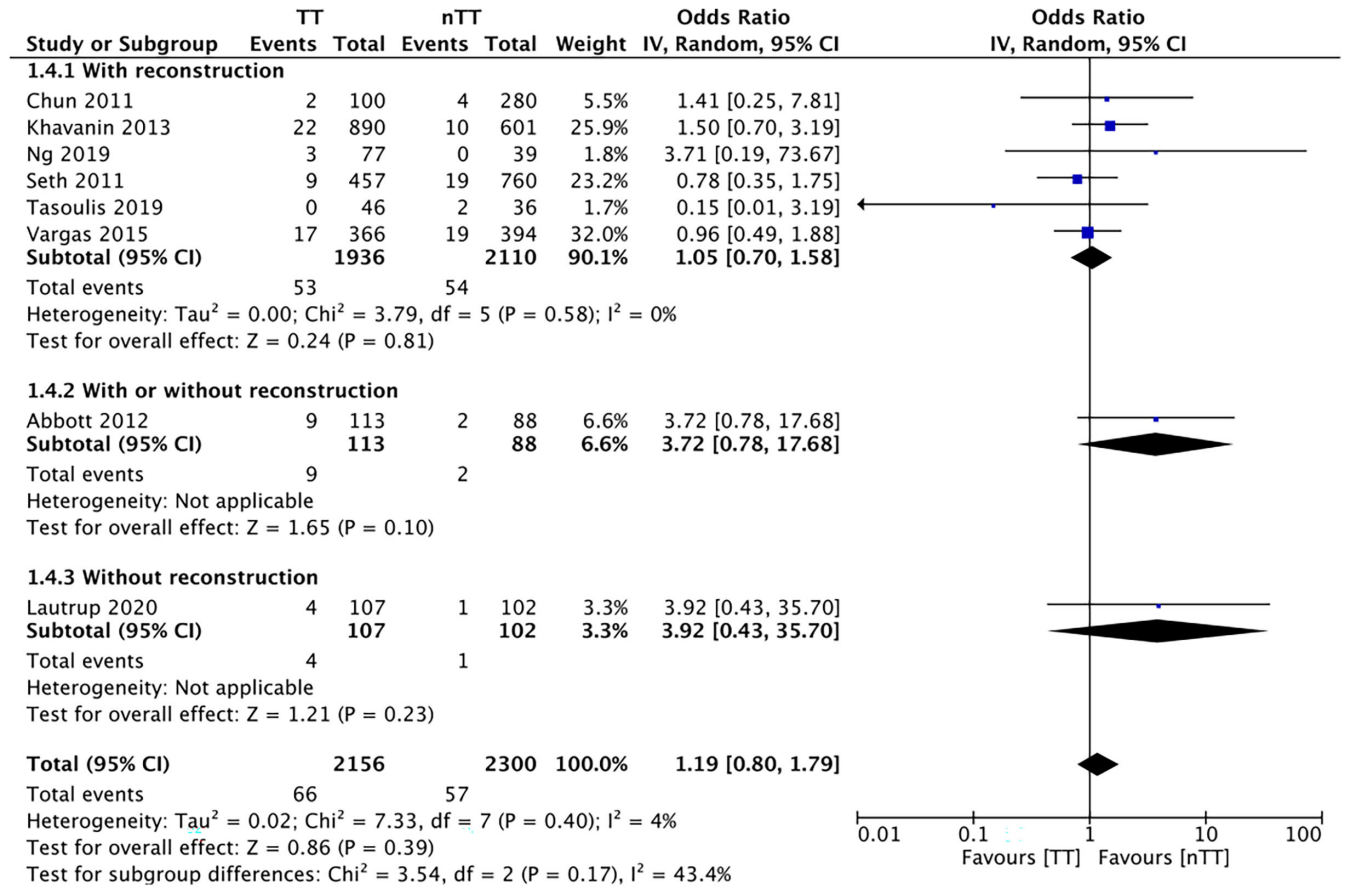
Forest plot for hematoma in sub-group analysis based on immediate reconstruction TT, tumescent group; nTT, non-tumescent group; IV, inverse variance; OR, odds ratio.

**Figure 6:**
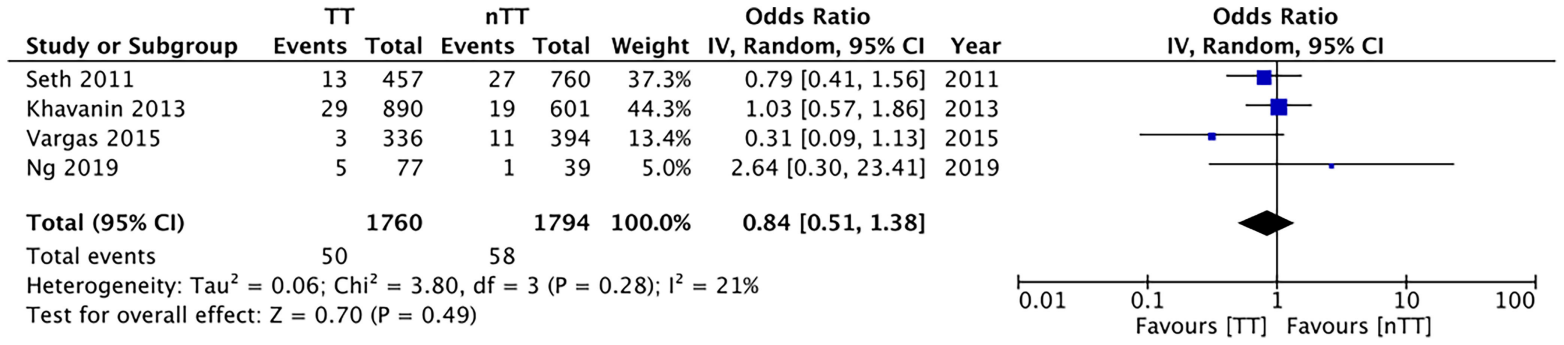
Forest plot for seroma with sub-group analysis based on immediate reconstruction TT, tumescent group; nTT, non-tumescent group; IV, inverse variance; OR, odds ratio.

**Figure 7:**
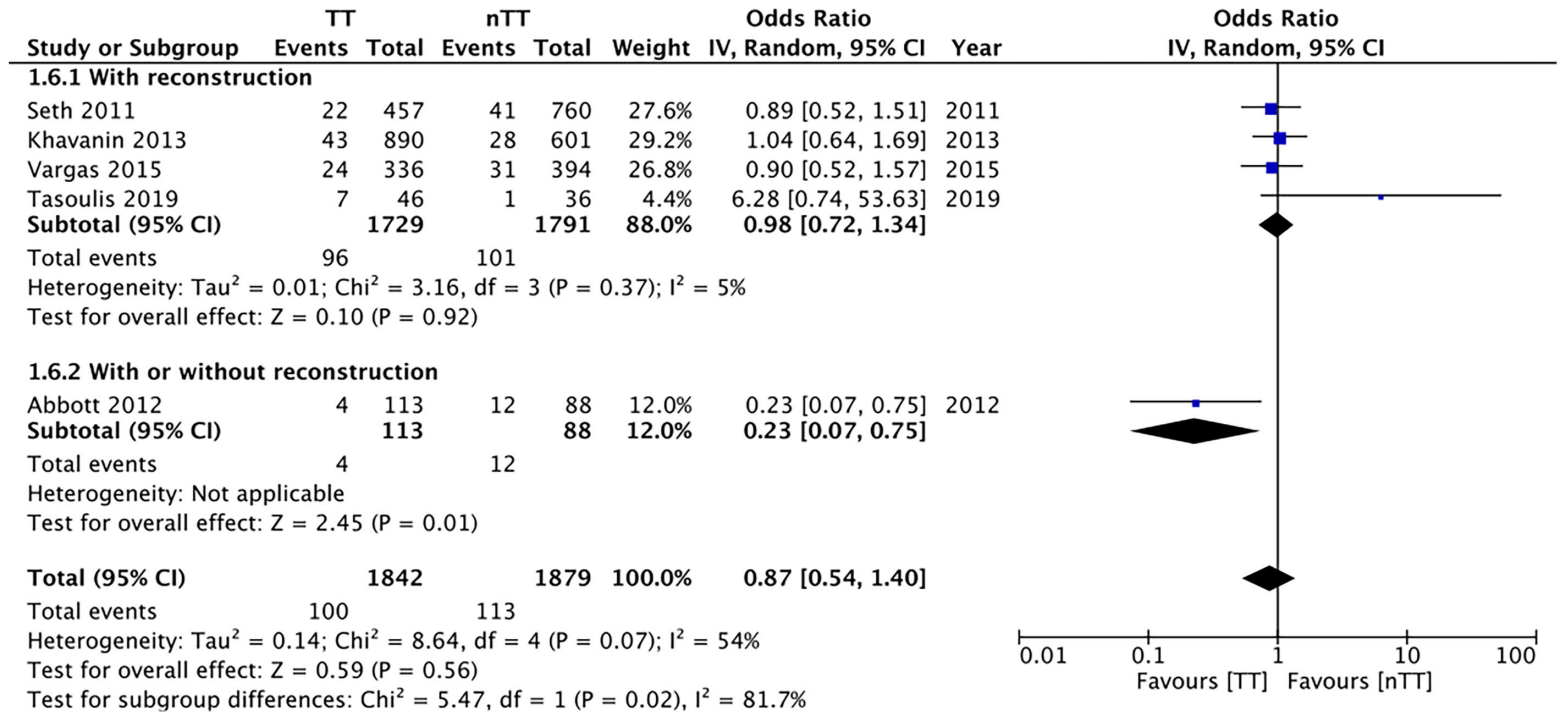
Forest plot for infections in sub-group analysis based on immediate reconstruction TT, tumescent group; nTT, non-tumescent group; IV, inverse variance; OR, odds ratio.

**Figure 8:**
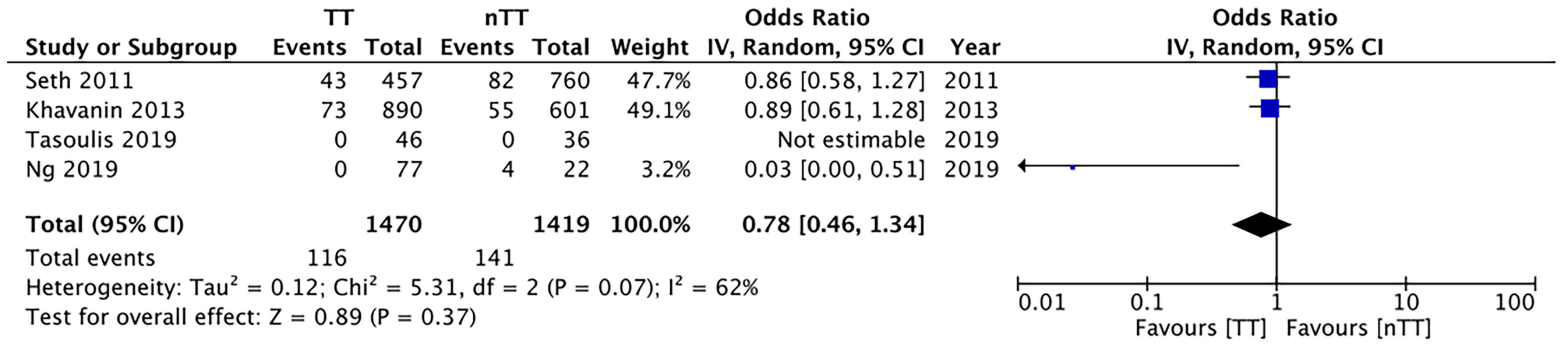
Forest plot for explantation or conversion to autologous reconstruction in patients undergoing immediate alloplastic reconstruction. TT, tumescent group; nTT, non-tumescent group; IV, inverse variance; OR, odds ratio.

We found no statistically significant differences in the incidence of hematoma requiring re-intervention between the tumescent and non-tumescent groups (OR, 1.19; 95% CI, 0.80 to 1.79; *I^2^ = *4%; *p*=0.39). The results were similar on the sub-group analysis based on the use of immediate reconstruction (**Figure 5**). A meta-analysis of studies using immediate reconstruction with mastectomy indicated no significant differences in the incidence of seroma between two study groups (OR, 0.84; 95% CI, 0.51 to 1.38; *I^2^ = *21%; *p*=0.49) (**Figure 6**). Similarly, we found a similar incidence of infections between the two groups (OR, 0.87; 95% CI, 0.54 to 1.40; *I^2^ = *54%; *p*=0.56) (**Figure 7**). And, our results were similar for all studies using immediate reconstruction; only in the study by Abott et al (21) did we find the incidence of infections to be significantly lower in the tumescent group than in the non-tumescent group (OR, 0.23; 95% CI, 0.07 to 0.75; *p*=0.01) (**Figure 7**). The analysis of studies using immediate alloplastic reconstruction (tissue expander or implant) revealed no statistically significant differences in the incidences of explantation or conversion to autogenous reconstruction between the two groups (OR, 0.78; 95% CI, 0.46 to 1.34; *I^2^ = *62%; *p*=0.37) (**Figure 8**).

**Figure 9:**
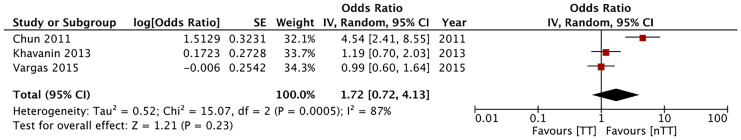
Forest plot of multivariable adjusted odds ratios for total complications and total skin necrosis. TT, tumescent group; nTT, non-tumescent group; IV, inverse variance; OR, odds ratio.

Multivariable-adjusted ORs on total skin necrosis were available from three studies. Our pooled analysis indicated similarities between the tumescent and non-tumescent groups (OR, 1.72; 95% CI, 0.72 to 4.13; *I^2^ = *87%; *p*=0.23) (**Figure 9**). Due to lack of data we could not complete this type of analysis for other outcomes.

### Quality Assessment

**Table 2** presents our quality assessment for the included studies. Blinding was not possible in the RCT due to the nature of the intervention. For all non-RCTs, we found a high risk of bias due to unadjusted confounding factors.

**Table 2:** Risk of bias in the studies included.

RCT						
Study	Random sequence generation	Allocation concealment	Blinding of participants and personnel	Blinding of outcome assessment	Incomplete outcome data	Selective reporting
Lautrup et al (15)	Low risk	Low risk	High risk	High risk	Low risk	Low risk
Retrospective studies						
Study	Selection of participants	Confounding variables	Intervention measurements	Blinding of outcome assessment	Incomplete outcome data	Selective outcome reporting
Chun et al (23)	Low risk	High risk	Low risk	High risk	Low risk	Low risk
Seth et al (22)	Low risk	High risk	Low risk	High risk	Low risk	Low risk
Abbott et al (21)	Low risk	High risk	Low risk	High risk	Low risk	Low risk
Khavanin et al (20)	Low risk	High risk	Low risk	High risk	Low risk	Low risk
Vargas et al (18)	Low risk	High risk	Low risk	High risk	Low risk	Low risk
Gipponi et al (17)	Low risk	High risk	Low risk	High risk	Low risk	Low risk
Ng et al (16)	Low risk	High risk	Low risk	High risk	Low risk	Low risk
Tasoulis et al (19)	Low risk	High risk	Low risk	High risk	Low risk	Low risk

RCT, randomized control trial.

## Discussion

The results of our updated systematic review and meta-analysis based mostly on non-RCTs indicate that the complication rates (skin necrosis, hematoma, seroma, and infections) may be similar for both the tumescent and non-tumescent dissection mastectomy techniques. The incidences of explantation or conversion to autogenous reconstruction were similar between the two dissection techniques in patients undergoing alloplastic reconstruction.

The optimal separation of the subcutaneous tissues containing the sub-dermal plexus from the gland parenchyma is essential to maximize flap survival during mastectomies. This dissection is also important from an oncological point of view; thick flaps may result in recurrence of malignancy in the remnant breast tissue, but thin flaps may lead to skin necrosis (18, 24). Electrocautery has been widely used as a conventional dissection technique in institutions worldwide. When compared to scalpel dissection, electrocautery is associated with less blood loss and a lack of cosmetic outcome or patient satisfaction score differences (25). However, the high temperatures needed for the electrocautery can cause significant ischemic lesions on the skin and subcutaneous tissues leading to wide areas of necrosis (17, 26, 27). The tumescent technique is thought to reduce the thermo-dispersion of the electrocautery thereby improving flap survival. However, concerns about the incidence of skin necrosis with the tumescent technique itself have also been raised. The vasoconstrictor effect of epinephrine and the compressive effect of the solution, which both reduce the blood loss are thought to contribute to reduced skin flap survival (23). In this context, our review presents important findings on the complications of these two techniques for surgeons carrying out mastectomies.

Our analysis revealed similar incidences for minor and major skin necrosis with both techniques. Skin necroses after mastectomies can be influenced by different confounding factors such as age, obesity, smoking, diabetes mellitus, and prior radiation therapy (11, 28, 29) that can be controlled for only in well-conducted RCTs to provide high-quality evidence. Unfortunately, only one RCT was available for inclusion in our review. For the remaining studies, the allocations were not randomized, and known and unknown patient characteristics differed between the study groups. Therefore, our results need to be interpreted with caution as the non-significant difference between the two techniques may not necessarily be due to intervention equivalence, but could have been caused by systematic differences between the groups themselves (30). While all non-RCTs in our analysis reported minimal differences between the two groups, none of them carried out propensity-score matching to adjust for baseline factors. Only three studies reported results of multivariable regression analysis for total skin necrosis. Our pooled analysis of such data indicated a similar incidence of total skin necrosis for both techniques. The use of immediate reconstruction, especially alloplastic reconstruction, is an important variable affecting immediate local complications (31). While most of the studies included used reconstruction in all the patients, two studies did not. Hence, we conducted a sub-group analysis including a single study in each group and obtained similar results. We further analyzed the incidence of explantation or conversion to autologous grafts in patients undergoing alloplastic reconstruction and the results of our meta-analysis demonstrated no adverse effect of the tumescent technique on the risk of explantation. However, it needs to be mentioned that the outcomes with tumescent technique can depend on many factors related to breast reconstruction namely the position of implant (pre-pectoral or retro-pectoral) and the use of mesh (synthetic or acellular). Traditionally, there has been a strong correlation between the use of tumescent technique and pre-pectoral implant placement and use of mesh due to advantages like shorter surgical time, reduced bleeding and easier dissection (32). More recently, tumescent technique has been used for retro-pectoral implant placement as well. Shimuzu et al (33) in a retrospective review of 35 patients undergoing awake breast augmentation with intercostal nerve blocks and tumescent technique have reported good outcomes with both pre-pectoral and retro-pectoral implant placement. Depending upon the position of implant, the tumescent solution needs to be injected either between the mammary gland and the pectoralis muscle or beneath the muscle in case of retro-pectoral implant placement. An important difference between the two is the amount of tumescent solution needed. In case of pre-pectoral implant placement, researchers have reported use of 400 to 700 ml of solution per breast while around 740 ml was needed for retro-pectoral implant placement (34). In our review, the included studies used various modalities of autologous and alloplastic reconstruction with differences in the use mesh and position of implant. Since outcomes were not reported separately for each modality of breast reconstruction, we were unable to discern evidence on the efficacy of tumescent technique for different methods of breast reconstruction. Additionally, it also needs to be pointed out that in recent times video-assisted and robot-assisted surgery is slowly gaining attention, especially for nipple-sparing mastectomy (35, 36). These minimally invasive techniques have proven to be safe, with low conversion rate to open surgery and acceptable complication rates. Lai et al (35) in a consensus statement on robotic-nipple sparing mastectomy have recommended the use of tumescent technique for development of the skin flap. Our review was, however, unable to assess the efficacy of tumescent technique for robotic surgeries due to lack of comparative data.

In the case of the tumescent technique, once the effect of the vasoconstrictor disappears, rebound bleeding and hematoma formation can ensue (15). Hematoma requiring re-intervention is a serious complication, we assessed its incidence in our review. Our meta-analysis revealed similar hematoma incidences with the use of the tumescent technique. We obtained similar results for seroma and infectious complications. As mentioned earlier, these outcomes could have been caused by several confounding factors like co-morbidities, axillary dissection, surgical technique, use of antibiotics, and local wound care, variables which were not controlled in retrospective studies (37, 38).

Our results differed from those in the previously published meta-analysis. Siotos et al (3) reported a significant increase in the risk of total skin necrosis (OR, 1.56; 95% CI, 1.04 to 2.35; *I^2^ = *71%; *p*=0.03), major skin necrosis (OR, 2.01; 95% CI, 1.29 to 3.14; *I^2^ = *29%; *p*=0.002), and minor skin necrosis (OR, 1.75; 95% CI, 1.06 to 2.90; *I^2^ = *0%; *p*=0.03) with the use of the tumescent technique. However, we found no such difference after our analysis and review. This can be attributed to the inclusion of three more studies in our analysis that provided a significant update. Furthermore, our review was strengthened by the additional analyses on the incidences of seroma and explantation, which had not been carried out in the previous study. To account for confounding factors and provide a comprehensive review, we pooled the data on multivariable-adjusted ORs.

The limitations of our review include the large number of retrospective studies in the analysis with their inherent bias and the fact that many studies had small sample sizes, which may have skewed our results. In addition, the studies included presented different types of surgical procedures and of reconstructions during the standard non-tumescent technique, and different follow-up durations creating methodological heterogeneity among them. Moreover, Seth et al (22) and Khavanin et al (20) reported data from the same institution with an overlap of four years. Also, complications with mastectomies can be influenced by the surgical skill and experience of the surgeons, and the impact of this factor on our results is difficult to assess. Lastly, we were unable to analyse oncological outcomes between tumescent and standard surgical techniques due to lack of data from include studies. Future studies should also report oncological outcomes in order to better assess the outcomes associated with the use of tumescent technique.

To conclude, the available evidence was of low-quality and derived mostly from non-randomized studies, but our analysis results suggest that the incidences of skin necrosis, hematoma, seroma, infection, and explantation between the tumescent and non-tumescent mastectomy techniques are similar. High-quality RCTs assessing the role of the tumescent technique with different reconstruction methods are needed to strengthen the evidence.

## Data Availability Statement

Publicly available datasets were analyzed in this study. This data can be found here: PubMed, Embase, BioMed Central, Ovid, and CENTRAL databases.

## Author Contributions

YY designed the project. JZ, XQ, and JF were involved in data collection and data analysis. YY prepared the manuscript. FS edit the manuscript. All authors read and approved the final manuscript.

## Conflict of Interest

The authors declare that the research was conducted in the absence of any commercial or financial relationships that could be construed as a potential conflict of interest.

## Publisher’s Note

All claims expressed in this article are solely those of the authors and do not necessarily represent those of their affiliated organizations, or those of the publisher, the editors and the reviewers. Any product that may be evaluated in this article, or claim that may be made by its manufacturer, is not guaranteed or endorsed by the publisher.
