# Supplementary material for: Complication Differences Between the Tumescent and Non-Tumescent Dissection Techniques for Mastectomy: A Meta-Analysis
Source: Front Oncol. 2022 Jan 10;11:648955. doi: 10.3389/fonc.2021.648955 (PMC8785857; doi:10.3389/fonc.2021.648955)
Supplement: Supplementary file 1 [file Table_1.docx]

Supplementary Table S1. Search strategy and PubMed database results

| **Search number** | **Query** | **Search Details** | **Results** |
| --- | --- | --- | --- |
| **1** | (breast surgery) AND (tumescent) | ("breast"[MeSH Terms] OR "breast"[All Fields] OR "breasts"[All Fields] OR "breast s"[All Fields]) AND ("surgery"[MeSH Subheading] OR "surgery"[All Fields] OR "surgical procedures, operative"[MeSH Terms] OR ("surgical"[All Fields] AND "procedures"[All Fields] AND "operative"[All Fields]) OR "operative surgical procedures"[All Fields] OR "general surgery"[MeSH Terms] OR ("general"[All Fields] AND "surgery"[All Fields]) OR "general surgery"[All Fields] OR "surgery s"[All Fields] OR "surgerys"[All Fields] OR "surgeries"[All Fields]) AND ("tumescence"[All Fields] OR "tumescent"[All Fields]) | 95 |
| **2** | (breast surgery) AND (hydrodissection) | ("breast"[MeSH Terms] OR "breast"[All Fields] OR "breasts"[All Fields] OR "breast s"[All Fields]) AND ("surgery"[MeSH Subheading] OR "surgery"[All Fields] OR "surgical procedures, operative"[MeSH Terms] OR ("surgical"[All Fields] AND "procedures"[All Fields] AND "operative"[All Fields]) OR "operative surgical procedures"[All Fields] OR "general surgery"[MeSH Terms] OR ("general"[All Fields] AND "surgery"[All Fields]) OR "general surgery"[All Fields] OR "surgery s"[All Fields] OR "surgerys"[All Fields] OR "surgeries"[All Fields]) AND "hydrodissection"[All Fields] | 22 |
| **3** | (mastectomy) AND (tumescent) | ("mastectomy"[MeSH Terms] OR "mastectomy"[All Fields] OR "mastectomies"[All Fields] OR "mastectomy, simple"[MeSH Terms] OR ("mastectomy"[All Fields] AND "simple"[All Fields]) OR "simple mastectomy"[All Fields]) AND ("tumescence"[All Fields] OR "tumescent"[All Fields]) | 36 |
| **4** | (mastectomy) AND (hydrodissection) | ("mastectomy"[MeSH Terms] OR "mastectomy"[All Fields] OR "mastectomies"[All Fields] OR "mastectomy, simple"[MeSH Terms] OR ("mastectomy"[All Fields] AND "simple"[All Fields]) OR "simple mastectomy"[All Fields]) AND "hydrodissection"[All Fields] | 10 |
| **5** | ((breast surgery) AND (epinephrine)) AND (lignocaine) | ("breast"[MeSH Terms] OR "breast"[All Fields] OR "breasts"[All Fields] OR "breast s"[All Fields]) AND ("surgery"[MeSH Subheading] OR "surgery"[All Fields] OR "surgical procedures, operative"[MeSH Terms] OR ("surgical"[All Fields] AND "procedures"[All Fields] AND "operative"[All Fields]) OR "operative surgical procedures"[All Fields] OR "general surgery"[MeSH Terms] OR ("general"[All Fields] AND "surgery"[All Fields]) OR "general surgery"[All Fields] OR "surgery s"[All Fields] OR "surgerys"[All Fields] OR "surgeries"[All Fields]) AND ("epinephrine"[MeSH Terms] OR "epinephrine"[All Fields] OR "adrenalin"[All Fields] OR "adrenaline"[All Fields] OR "epinephrin"[All Fields] OR "epinephrines"[All Fields]) AND ("lidocain"[All Fields] OR "lidocaine"[MeSH Terms] OR "lidocaine"[All Fields] OR "lignocaine"[All Fields] OR "lidocaine s"[All Fields] OR "lignocain"[All Fields]) | 58 |
| **6** | ((breast surgery) AND (epinephrine)) AND (local anesthetic) | ("breast"[MeSH Terms] OR "breast"[All Fields] OR "breasts"[All Fields] OR "breast s"[All Fields]) AND ("surgery"[MeSH Subheading] OR "surgery"[All Fields] OR "surgical procedures, operative"[MeSH Terms] OR ("surgical"[All Fields] AND "procedures"[All Fields] AND "operative"[All Fields]) OR "operative surgical procedures"[All Fields] OR "general surgery"[MeSH Terms] OR ("general"[All Fields] AND "surgery"[All Fields]) OR "general surgery"[All Fields] OR "surgery s"[All Fields] OR "surgerys"[All Fields] OR "surgeries"[All Fields]) AND ("epinephrine"[MeSH Terms] OR "epinephrine"[All Fields] OR "adrenalin"[All Fields] OR "adrenaline"[All Fields] OR "epinephrin"[All Fields] OR "epinephrines"[All Fields]) AND ("anesthetics local"[Pharmacological Action] OR "anesthetics, local"[MeSH Terms] OR ("anesthetics"[All Fields] AND "local"[All Fields]) OR "local anesthetics"[All Fields] OR ("local"[All Fields] AND "anesthetic"[All Fields]) OR "local anesthetic"[All Fields] OR "anesthesia, local"[MeSH Terms] OR ("anesthesia"[All Fields] AND "local"[All Fields]) OR "local anesthesia"[All Fields] OR ("local"[All Fields] AND "anesthetic"[All Fields])) | 83 |
| **7** | ((mastectomy) AND (epinephrine)) AND (local anesthetic) | ("mastectomy"[MeSH Terms] OR "mastectomy"[All Fields] OR "mastectomies"[All Fields] OR "mastectomy, simple"[MeSH Terms] OR ("mastectomy"[All Fields] AND "simple"[All Fields]) OR "simple mastectomy"[All Fields]) AND ("epinephrine"[MeSH Terms] OR "epinephrine"[All Fields] OR "adrenalin"[All Fields] OR "adrenaline"[All Fields] OR "epinephrin"[All Fields] OR "epinephrines"[All Fields]) AND ("anesthetics local"[Pharmacological Action] OR "anesthetics, local"[MeSH Terms] OR ("anesthetics"[All Fields] AND "local"[All Fields]) OR "local anesthetics"[All Fields] OR ("local"[All Fields] AND "anesthetic"[All Fields]) OR "local anesthetic"[All Fields] OR "anesthesia, local"[MeSH Terms] OR ("anesthesia"[All Fields] AND "local"[All Fields]) OR "local anesthesia"[All Fields] OR ("local"[All Fields] AND "anesthetic"[All Fields])) | 25 |
| **8** | ((mastectomy) AND (epinephrine)) AND (lignocaine) | ("mastectomy"[MeSH Terms] OR "mastectomy"[All Fields] OR "mastectomies"[All Fields] OR "mastectomy, simple"[MeSH Terms] OR ("mastectomy"[All Fields] AND "simple"[All Fields]) OR "simple mastectomy"[All Fields]) AND ("epinephrine"[MeSH Terms] OR "epinephrine"[All Fields] OR "adrenalin"[All Fields] OR "adrenaline"[All Fields] OR "epinephrin"[All Fields] OR "epinephrines"[All Fields]) AND ("lidocain"[All Fields] OR "lidocaine"[MeSH Terms] OR "lidocaine"[All Fields] OR "lignocaine"[All Fields] OR "lidocaine s"[All Fields] OR "lignocain"[All Fields]) | 15 |
